# Comparison of Two Tonometers in the Evaluation of 24-Hour Intraocular Pressure and Mean Ocular Perfusion Pressure in Patients with Thyroid-Associated Ophthalmopathy

**DOI:** 10.1155/2022/8628362

**Published:** 2022-04-22

**Authors:** Weijie Liu, Yao Zhou, Xiaozhou Hu, Haochen Jin, Jie Ye, Mingna Xu, Zihui Liu, Wencan Wu, Yunhai Tu

**Affiliations:** The Eye Hospital of Wenzhou Medical University, Wenzhou, China

## Abstract

**Purpose:**

The aim of the study is to compare a non-contact tonometer (NCT) and goldmann applannation tonometer (GAT) in the evaluation of intraocular pressure (IOP) and mean ocular perfusion pressure (MOPP) in patients with thyroid-associated ophthalmopathy (TAO).

**Methods:**

In this study, a total of 30 patients (16 females and 14 males) were recruited. All patients underwent a routine ophthalmic assessment and their medical history was acquired. Clinical assessment included the 24-hour measurement of intraocular pressure and blood pressure, an orbital computed tomography (CT) scan, and a visual field (VF）test. Patients were divided into two groups according to their visual field test results: a defect group with mean deviation (MD) of visual field −2 dB or lower and a normal group with MD over −2 dB.

**Results:**

Bland–Altman's analysis showed similar results of IOP at every time point and revealed an agreement of mean IOP between the two tonometers (the deviation in the mean IOP between the two tonometers was 1 mmHg, with 95% limits of agreement of 8.8 to −6.8 mmHg). The 24-hour MOPP SD value in NCT (2.28) and GAT (1.77) showed that the two instruments had the same diagnostic efficacy (100% sensitivity, 95.8% specificity). The areas under the receiver operator characteristic (ROC) curve of the 24-hour mean ocular perfusion pressure (MOPP) SD (GAT: 0.778, NCT: 0.713; *z* = 0.669, *P*=0.504), 24-hour MOPP fluctuation (GAT:0.683, NCT:0.757; *z* = 0.963, *P*=0.336) measured by GAT and NCT had no significant difference between the two tonometers.

**Conclusions:**

The measurement of IOPs, MOPPs, and their diagnostic efficacy of visual field defect showed consistency between NCT and GAT. The study highlights the importance of monitoring the 24-hour MOPP and IOP in TAO patients. Furthermore, it suggests that the less invasive NCT can replace GAT as a long-term monitoring device in TAO patients.

## 1. Introduction

Dysthyroid optic neuropathy (DON) is the most severe complication of thyroid-associated ophthalmopathy (TAO) [1]. Early clinical signs include abnormal color vision and contrast sensitivity loss, whereas in later stages of the disease, it can lead to irreversible loss of vision or even blindness. The exact pathogenesis of the condition is still unclear, and further research is needed to assist in its early diagnosis and treatment.

Normal blood perfusion is vital in maintaining the structure and function of the surrounding tissue. Therefore, blood perfusion (BP) disorder caused by abnormal blood pressure fluctuation contributes to damage in organs such as the heart and brain or aggravates pre-existing tissue damage [2, 3]. Ocular perfusion pressure is determined by the difference between mean arterial pressure and intraocular pressure（IOP）, which are crucial to keeping the eye's anatomy and proper function [4]. The relationship between ocular perfusion disorder and optic nerve injury has received increasing attention in optic neuropathy.

Studies have shown that the 24-hour IOP and MOPP fluctuation could aggravate optic neuropathy in glaucoma [5–8]. In TAO patients, our early results indicated a significant difference in the 24-hour MOPP fluctuation amongst the TAO patients with and without visual field defects, and the 24-hour MOPP fluctuation was strongly correlated with visual field defects [9]. Those results suggested that the 24-hour MOPP fluctuation may be related to the pathogenesis of DON. Therefore, monitoring of the 24-hour MOPP in TAO patients can be of particular clinical significance.

Although Goldmann's applannation tonometer (GAT) has always been considered the gold standard for measuring IOP [10], it requires direct contact with the cornea and has the risk of corneal injury and secondary infection [11]. In TAO patients, where lagophthalmos or exposure keratitis are common [12], repeated GAT measurements over the day can further increase the risk. In this work, we compared IOP values measured by GAT and NCT in TAO patients to evaluate their differences in 24-hour MOPP monitoring and to explore the possibility of using NCT instead of GAT to monitor 24-hour MOPP in TAO patients.

## 2. Methods

### 2.1. Participants

The participants in this study were patients diagnosed with TAO using Bartley's diagnostic criteria at The Eye Hospital of Wenzhou Medical University from January 2018 to March 2019. The study adhered to the tenets of the Declaration of Helsinki and was approved by the local ethics committee (Medical Ethics Committee, the Eye Hospital of Wenzhou Medical University, Wenzhou, China). Informed consent was obtained from all patients. All patients underwent a review of their medical history, 24-hour blood pressure examination (the Mercury Sphygmomanometer; Yuwell, China), and a comprehensive eye exam including best-corrected visual acuity test, exophthalmos measurement, slit-lamp microscopic examination, dilated fundoscopic examination, 24-hour Goldmann tonometry (the Applanation Tonometer AT-900; Hagg-streit Koniz, Switzerland), 24-hour NCT examination (the Full Auto Tonometer TX-20; Canon, Japan), and visual field (VF) test (Humphery Field Analyzer 750I; SITA standard algorithms with 24–2 program; Zesis, Germany). The VF test was repeated to ensure the reliability of the measurement (defined as a false-positive error <15%, false-negative error <15%, and fixation loss <20%).

Patients with one or more of the following conditions were excluded from the study: (1)had any other oculopathy that affected visual function and eye movement, (2) had undergone orbital wall decompression surgery, ocular radiation therapy, or other eye surgery, (3) in the acute stage or patients with lagophthalmos (4) do not accept 24-hour IOP test or blood pressure monitoring, (5) were undergoing treatment to control IOP and blood pressure, and (6) a history of diabetes and hypertension.

### 2.2. Patient Classification

Patients were classified into two groups based on the mean deviation (MD value) from a visual field test: a visual field defect group (MD < −2 dB) and a normal group (MD ≥ −2 dB).

### 2.3. 24-Hour IOP and BP Monitoring

Patients were admitted to the hospital to monitor BP and IOP over a 24-hour period. To avoid damage to the cornea and disturbing their sleep, measurements were taken at 05:00, 07:00, 10:00, 14:00, 18:: 00, and 22:00. Systolic and diastolic BP (SBP and DBP, respectively) were measured with a brachial sphygmomanometer on the upper left arm with the patient sitting upright for at least 5 min to keep calm and reduce possible errors prior to the measurement. IOP was measured with GAT after the NCT test, and the measurement location was required to be the center of cornea. Both IOP tests were performed with the patient seated and used neutral position of the neck to minimize the measurement error caused by different body positions [13, 14]; patients were asked to refrain from any physical activities like running, etc. that could affect BP or IOP during admission [15]. Meals were provided at 06:30, 11:30, and 19:30 and did not include any alcohol or caffeine [15]. The abovementioned measurement process was completed by an ophthalmologist in training. The average value of three consecutive measurements was taken at each time point.

### 2.4. Calculation of IOP and BP

Perfusion pressure (PP) was defined as the difference between SBP and DBP. Mean arterial pressure (MAP) was defined as the average pressure throughout the cardiac cycle and represented an individual's perfusion pressure and it was calculated by the formula: MAP = DBP + 1/3 (SBP − DBP) [16]. MOPP was measured with the patient seated in an upright position and calculated by the formula MOPP = 2/3 MAP − IOP [16]. The 24-hour MOPP fluctuation was defined as the difference between the MOPP maximum measurement and MOPP minimum measurement. The MOPP standard deviation (SD) was also calculated from the MOPP measurements in the 24-hour period. Similarly, 24-hour IOP fluctuation and standard deviation were calculated.

### 2.5. Statistical Analysis

Statistical analysis was performed using SPSS 25.0 (SPSS Inc, Chicago, Illinois, USA) and MedCalc v.18 (MedCalc Software bvba, Ostend, Belgium). Descriptive statistics (values for categorical variables and mean and standard deviation for continuous variables) were initially evaluated. The independent-samples *t*-test was used to study IOP, PP, and OPP values among the two groups. The Chi-square (*χ*^2^) test was used to detect the difference between the two sample rates, and the Pearson correlation coefficient was used to determine the correlation between the visual field test result and parameters BP, IOP, and MOPP. A Bland–Altman analysis was carried out to investigate NCT and GAT's agreement in IOP measurement. Finally, ROC curve analysis was used to compare the diagnostic efficacy of 24-hour MOPP measured by the two tonometers in TAO patients with and without VF defect. (*P* < 0.05 for statistical significance).

## 3. Results

A total of 30 patients (60 eyes) were enrolled in this study from January 2018 to March 2019, including 16 females and 14 males. The average age was 47.97 ± 10.93 years old (26–67 years old). Among the 60 eyes, 36 eyes were classified as VF defected, and 24 eyes as VF normals. MD value of the left and right eye was −5.5 ± 7.6 dB and −6.5 ± 7.6 dB, respectively, and the differences between the two groups were not statistically significant (*P*=0.562). All relevant information for the patients is shown in Table 1.

### 3.1. IOP Measurement Using NCT and GAT

**Figure 1:**
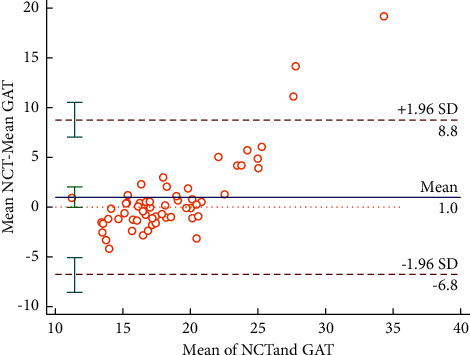
Bland–Altman's analysis of the mean IOP between NCT and GAT.

**Figure 2:**
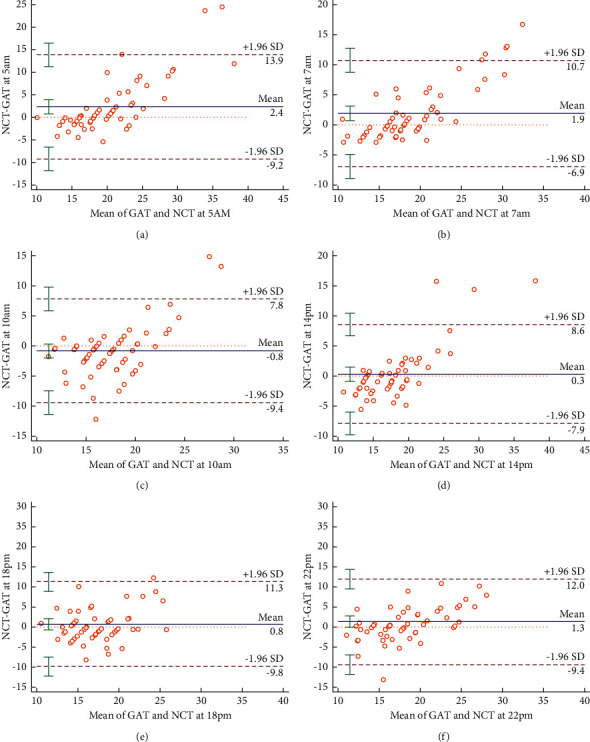
(a)–(f). Bland–Altman's analysis of IOP at every time point between NCT and GAT.

There was no statistical difference between the 24-hour IOP measured by NCT (18.93 ± 5.95 mmHg) and GAT (17.94 ± 2.77 mmHg) (*P*=0.059). The Bland–Altman's analysis revealed a bias of the mean IOP between NCT and GAT of 1 mmHg, with 95% limits of agreement of 8.8 to −6.8 mmHg (Figure 1). The Bland–Altman analysis showed similar results at every time point (Figures 2(a)–2(f)).

### 3.2. Relationship between IOP/MOPP and VF in TAO Patients

The difference between IOP/MOPP measured by the two tonometers at different time points and between the visual defect group and the normal group is shown in Table 2. No significant difference was found in the mean, the standard deviation (SD), 24-hour fluctuation of IOP, and mean of MOPP between the defect and the normal group for either instrument. However, the SD and 24-hour fluctuation of MOPP were found to be statistically different between the two groups for either of the instruments (*P* < 0.05). Additionally, the differences in the 24-hour MOPP SD and fluctuation values between the two groups strongly correlated with MD values of VF (Table 3.).

### 3.3. ROC Curve Analysis

**Figure 3:**
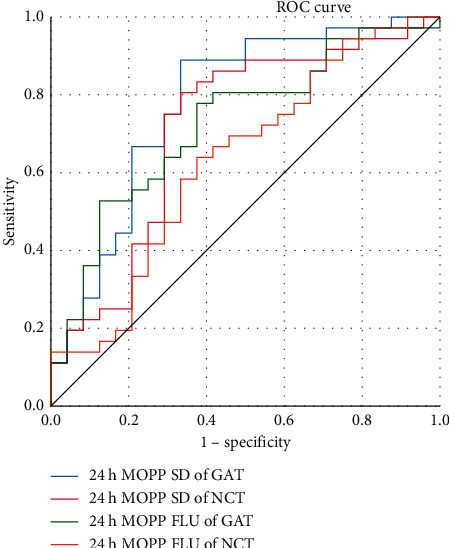
ROC curve for the 24-hour MOPP SD and fluctuation measured by the NCT and GAT. (SD: standard).

ROC curve analysis was performed to compare the diagnostic efficacy of 24-hour MOPP measured by the two tonometers in TAO patients with and without VF defect. The cut off values of 24-hour MOPP SD measured by GAT and NCT were 4.132 and 5.713, respectively. The areas under the ROC curve of the 24-hour MOPP SD measured by GAT and NCT were 0.778 and 0.713, respectively, and there was no significant difference between the two tonometers (*z* = 0.669, *P*=0.504). The cut off values of 24-hour MOPP fluctuation measured by GAT and NCT were 6.546 and 7.905, respectively. The areas under the ROC curve of the 24-hour MOPP fluctuation values measured by GAT and NCT were 0.683 and 0.757, respectively, and, similarly to SD, there was no significant difference between the two tonometers (*z* = 0.963, *P*=0.336). The 24-hour MOPP SD value in NCT (2.28) and GAT (1.77) had the same diagnostic efficacy (100% sensitivity, 95.8% specificity) (Figure 3).

## 4. Discussion

In this study, two different tonometric instruments, namely, NCT and GAT, were compared for the evaluation of 24-hour IOP and MOPP in TAO patients. The results indicated that although there were some discrepancies in the measurements between the two tonometers, the most of the IOP data at every time point and the 24-hour mean IOP values measured by NCT and GAT were in agreement. There was no significant difference in the mean, fluctuation, and SD value of 24-hour IOP between TAO patients with and without VF defect, with either instrument. However, the fluctuation and SD of 24-hour MOPP were significantly different and found to correlate to the MD of the visual field test. The ROC curve analysis revealed that there was no significant difference in the diagnostic efficacy for the 24-hour MOPP SD value and the fluctuation value between NCT and GAT in TAO patients for either of the groups (normal and defect).

In previous studies, TAO patients showed increased IOP, ranging between 4.8% and 24% [17–19]. This increase was attributed to the congestion of orbital venous drainage [20], the abnormal structure of trabecular meshwork, the direction of gaze, or the extraocular movement restriction caused by enlarged extraocular muscles [16, 17]. In our study, the mean 24-hour IOP measured by the two tonometers was below 21 mmHg, but IOP values greater than 21 mmHg were observed, in line with previous studies [9, 21]. Although there was no significant difference in the mean, fluctuation, and SD of 24-hour IOP between the patients with and without VF defect, the mean and the fluctuation of 24-hour IOP was increased in patients with VF defect. Parekh et al. [22], using a contact lens sensor to monitor the 24-hour IOP, found that in TAO patients, the peak IOP occurred at 6:30am, whereas, in glaucoma patients, the IOP peak usually appeared at 2:30–3:00am It is believed that this peak may be a risk factor for glaucoma [23]. In our study, there was a significant difference in the IOP measured by GAT between the TAO patients with and without VF defect at 10pm (Table 3.), which is in accordance with the behavior of IOP in glaucoma patients and shows that high nocturnal IOP may also be a risk factor for DON. Therefore, 24-hour intraocular pressure monitoring, especially at night, is crucial for TAO patients.

Previous studies suggested that NCT showed higher readings of IOP compared to GAT. However, Tonnu et al. [10] found that NCT misreads IOP at the extremes; NCT underestimates IOP at low values and overestimates IOP at high values, which was also confirmed in other studies [24]. In our study, Bland–Altman's analysis showed that the two tonometers agreed in the normal IOP range. However, when the average IOP was high at different time points, NCT measurements were also higher. Similarly, when the average IOP was low, the value measured by NCT was lower, in line with previous studies [9, 21]. In this study, NCT measurements of individual patients with TAO showed that IOP was higher than 30 mmHg, whereas GAT measurements were lower than 21 mmHg, to which clinicians should pay great attention.

Lately, the development of VF defects in glaucoma patients due to an abnormal ocular perfusion pressure such as hypotension, nocturnal hypotension, and low MOPP [23, 25], and the 24-hour fluctuation of MOPP has been the subject of several studies [26, 27]. In a four-year follow-up in 101 normal-tension glaucomas (NTG) patients, significant differences were reported between progressors and nonprogressors in nocturnal MAP and MOPP fluctuations. The 24-hour MAP and MOPP fluctuation were strongly correlated with the progression of the VF defects. The Cox proportional hazards model also indicated that the progression of VF defects was associated with the 24-hour MOPP fluctuation [28]. In past studies by our group, a significant difference in the 24-hour MOPP fluctuation between the TAO patients with and without visual field defect and the 24-hour MOPP fluctuation negatively correlated with MD [9]. This result is consistent with previous glaucoma studies, suggesting that abnormal fluctuations in the 24-hour MOPP may lead to visual field defects in TAO patients. In this study, the fluctuation of 24-hour MOPP measured by two tonometers was statistically significant between the TAO patienets with and without VF defect, and both showed a significant negative correlation with MD of VF. The latter suggests that the monitoring of 24-hour MOPP fluctuation in TAO patients is of great clinical importance. In our study, ROC curve analysis showed that the diagnostic accuracy of NCT and GAT was the same in the 24-hour MOPP SD value in TAO patients with or without VF defect, and the areas under the ROC curve of the 24-hour MOPP SD, 24-hour MOPP fluctuation measured by GAT and NCT had no significant difference.

There are also some shortcomings in this study. Firstly, only a few IOP measurements were obtained within 24 hours. Considering that frequent GAT measurement may damage the cornea, and after a careful review of the literature [22], we found that the peak and valley values were observed by measuring the specific time points in TAO patients, so we chose the six time points used in the paper. Secondly, the sample size is limited, and there is no longitudinal study to verify the correlation between VF defect and 24-hour MOPP fluctuation. Thirdly, the interval for IOP measuring was long, so there might be some fluctuations in the intervals. Finally, we did not include the normal group of subjects and only conducted comparison and analysis among patients. In future studies, we will further verify the consistency of NCT and GAT by expanding the sample size and increase the time points of using NCT alone to monitor 24 h IOP.

## 5. Conclusions

In conclusion, it is of great clinical significance to strengthen the monitoring of 24-hour IOP and MOPP in TAO patients. Although NCT has some disadvantages, it can still be used as a long-term monitoring tool for the 24-hour MOPP and IOP monitoring in TAO patients.

## Figures and Tables

**Table 1:** Basic message of two groups.

	VF defect (*n* = 36)	VF normal (*n* = 24)	*P* value
Age	49.58 ± 11.41	45.54 ± 10.12	0.157
M/F	13/23	14/10	0.188
Exophthalmos	18.62 ± 2.43	19.70 ± 2.74	0.078
CAS score	1.77 ± 1.31	1.72 ± 1.19	0.871
MD	−8.74 ± 6.87	−1.54 ± 0.74	＜0.001^*∗∗*^
T3	2.15 ± 0.86	1.97 ± 0.64	0.394
T4	129.01 ± 42.58	125.68 ± 20.43	0.724
FT3	6.45 ± 4.27	5.56 ± 2.32	0.355
FT4	22.67 ± 15.19	19.62 ± 4.17	0.344
TSH	1.98 ± 4.40	4.13 ± 5.54	0.113

^*∗∗*^ *P* < 0.01; VF = visual field MD: mean defect, T3: triiodothyronine, FT3: free triiodothyronine, T4: serum total thyroxine, FT4: serum-free thyroxine, TSH: thyroid-stimulating hormone.

**Table 2:** IOP measured by the two tonometers at different time points between the groups with and without visual field defect.

		VF defect	VF normal	*P* value
5am	GAT	19.80 ± 4.21	18.00 ± 2.53	0.065
	NCT	21.95 ± 8.34	20.68 ± 7.97	0.557
7am	GAT	18.50 ± 3.83	17.66 ± 3.00	0.374
	NCT	20.55 ± 7.43	19.41 ± 6.62	0.538
10am	GAT	17.80 ± 3.27	18.04 ± 2.29	0.774
	NCT	17.00 ± 5.25	17.28 ± 5.30	0.84
2pm	GAT	17.94 ± 3.60	17.33 ± 2.66	0.447
	NCT	18.57 ± 6.98	17.24 ± 5.20	0.402
6pm	GAT	16.66 ± 3.31	17.77 ± 3.34	0.214
	NCT	18.68 ± 6.73	16.63 ± 4.59	0.167
10pm	GAT	18.63 ± 3.84	16.37 ± 2.94	0.011^*∗*^
	NCT	20.15 ± 7.62	17.37 ± 5.48	0.105

^*∗*^ *P* < 0.5.

**Table 3:** Relationship between 24-hour IOP/MOPP measured by the two tonometers and the MD value of VF.

			VF defect	VF normal	*P* value	Relation to MD	
						*R*	*P*
IOP	Mean	GAT	18.22 ± 3.15	17.51 ± 2.08	0.347	0.033	0.807
		NCT	19.48 ± 6.40	18.10 ± 5.22	0.383	0.017	0.898
	SD	GAT	1.94 ± 1.30	1.81 ± 0.98	0.682	−0.032	0.812
		NCT	3.42 ± 1.66	3.08 ± 1.68	0.437	0.169	0.204
	FLU	GAT	5.03 ± 3.22	4.52 ± 2.23	0.505	−0.05	0.71
		NCT	9.11 ± 4.42	7.97 ± 4.41	0.335	0.127	0.343

MOPP	Mean	GAT	58.52 ± 7.14	58.01 ± 8.03	0.798	−0.143	0.285
		NCT	57.19 ± 9.54	57.75 ± 9.10	0.824	−0.095	0.48
	SD	GAT	5.89 ± 1.98	4.07 ± 1.76	0.001^*∗∗*^	−0.494	<0.001^*∗∗*^
		NCT	6.99 ± 2.05	5.50 ± 1.86	0.006^*∗∗*^	−0.285	0.03^*∗*^
	FLU	GAT	15.57 ± 5.79	10.81 ± 4.64	0.001^*∗∗*^	−0.524	<0.001^*∗∗*^
		NCT	18.44 ± 5.78	14.63 ± 4.85	0.01^*∗*^	−0.339	0.009^*∗∗*^

^*∗*^ *P* < 0.05, ^*∗∗*^*P* < 0.01; IOP: intraocular pressure and SD: standard, FLU: fluctuation.

## Data Availability

All data generated or analyzed during this study are included in this article and its supplementary material files. Further enquiries can be directed to the corresponding author.
